# Case report: Diagnosis of apical hypertrophic cardiomyopathy that escaped clinical and echocardiographic investigations for twenty years: Reasons and clinical implications

**DOI:** 10.3389/fcvm.2023.1157599

**Published:** 2023-04-24

**Authors:** Carlo Caiati, Alessandro Stanca, Mario Erminio Lepera

**Affiliations:** Unit of Cardiovascular Diseases, Department of Interdisciplinary Medicine, University of Bari “Aldo Moro”, Bari, Italy

**Keywords:** apical hypertrophic cardiomyopathy, contrast echocardiography, heart failure, LV diastolic dysfunction, Doppler echocardiography

## Abstract

**Background:**

Apical hypertrophic cardiomyopathy (ApHCM) is a rare form of hypertrophic cardiomyopathy which predominantly affects the apex of the left ventricle. The diagnosis can be challenging due to several factors, ranging from no typical clinical and electrocardiogram (EKG) findings to potential difficulties in executing and interpreting the echocardiographic examination.

**Case presentation:**

We report the case of an 84-year-old woman who came to our echo-lab to undergo a routine echocardiogram. She had a history of permanent atrial fibrillation, paced rhythm and previous episodes of heart failure (HF), allegedly explained by a diagnosis of hypertensive heart disease that had been confirmed many times over the previous 20 years. The clinical examination and the EKG were unremarkable. The echocardiographic images were poor quality. But a senior cardiologist, expert in imaging and echocardiography, noted the lack of delineation of the endocardial border of the left ventricular (LV) apex region. Contrast echocardiography was performed and severe apical hypertrophy discovered.

**Conclusion:**

ApHCM can be a challenging diagnosis. Contrast echocardiography must always be applied in cases of poor delineation of the LV apical endocardial border at baseline echocardiography. Timely detection and appropriate lifestyle intervention might slow the development of LV hypertrophy, and possibly minimize and delay heart failure (HF) related symptoms and arrhythmias. The prognosis remains relatively benign during long term follow-up.

## Introduction

Apical hypertrophic cardiomyopathy (ApHCM) is a form of hypertrophic cardiomyopathy, more frequently found in Asian population (up to 25% of all HCM cases) (1, 2). The prognosis is in general less severe than that of a classical HCM (1). However the complications rate has been reported at 30% being the myocardial infarction and atrial fibrillation the most frequent (1, 2), although other less frequent complications have been reported like left ventricular (LV) apical aneurism, embolic events, ventricular fibrillation, congestive heart failure (3). Although the disease is genetically transmitted, the precise timing of diagnosis can be important since external factors can fuel the progression of the disease, strongly modulating its phenotypic expression (4, 5).

The diagnosis is based on clinical examinations, electrocardiogram (EKG) and echocardiography but each of these approaches can present limitations to such an extent that diagnosis can be missed as in our case for a long time. However critical interpretation of both the clinical symptoms and the echocardiogram can suggest the use of contrast echocardiography that can appropriately reveal the left ventricular apical abnormality. This is illustrated in the reported clinical case.

## Clinical case

### History

An 84-year-old woman with a history of obesity, hypertension, dyslipidaemia, carotid atherosclerosis and chronic obstructive pulmonary disease, presented to our echo-lab to undergo a routine echocardiogram. At the time, she had no complaints of chest pain, palpitations and dyspnoea at rest. There was no family history of sudden death, congestive heart failure or cardiomyopathy.

Twenty years before (in 2003) at the age of 64, after a syncopal episode, she had been implanted with a bicameral pacemaker for alleged sinoatrial node dysfunction; 9 years later the depleted battery was replaced.

About 15 years ago, (several years after pace-maker implantation) she was diagnosed with permanent atrial fibrillation and had since been on direct-acting oral anticoagulants.

In the last three years, she complained of dyspnoea following normal and less than normal physical activity (New York Heart Association [NYHA] class II-III). She also had limited mobility, due to back pain caused by multiple vertebral collapses, that further limited her physical capacity during effort. Therefore, the NYHA class was probably imprecise.

In 2020, a Holter EKG examination to verify pacemaker function identified an asymptomatic horizontal-down sloping ST-segment depression, suspicious for silent ischemia; for this reason she underwent coronary computed tomography angiography, that showed diffuse plaque involving the left anterior descending coronary artery (LAD), with >50% maximal segmental lumen narrowing. In the same year, she underwent coronary angiography; however, this did not confirm the critical LAD stenosis by Coronary Computed Angiography, so ruling out any significant coronary narrowing. Since intravascular ultrasound and coronary flow reserve were not assessed, diffuse coronary atherosclerosis could not be surely ruled out (6).

In 2022, the patient was admitted to an Internal Medicine ward, complaining of persistent fever lasting for several days and a confusional status. During hospitalization, a chest x-ray was performed which detected a consolidation in the right lung associated with an increase in inflammatory markers (C reactive protein = 17.8 mg/L), for which effective antibiotic therapy was initiated. Moreover, mild hyponatremia (129 mEq/L) was also observed and corrective treatment was administered. At discharge, a diagnosis of worsening heart failure, complicated by an acute bronchopneumonia episode, was made, although no clear explanation for this “chronic” heart failure was given. Increased dosage (50 mg/die) of a loop diuretic (furosemide) was prescribed.

Over the last twenty years, the patient had undergone several follow-up echocardiograms at qualified Hospitals, all in agreement as to the diagnosis of hypertensive heart disease.

**Figure 1:**
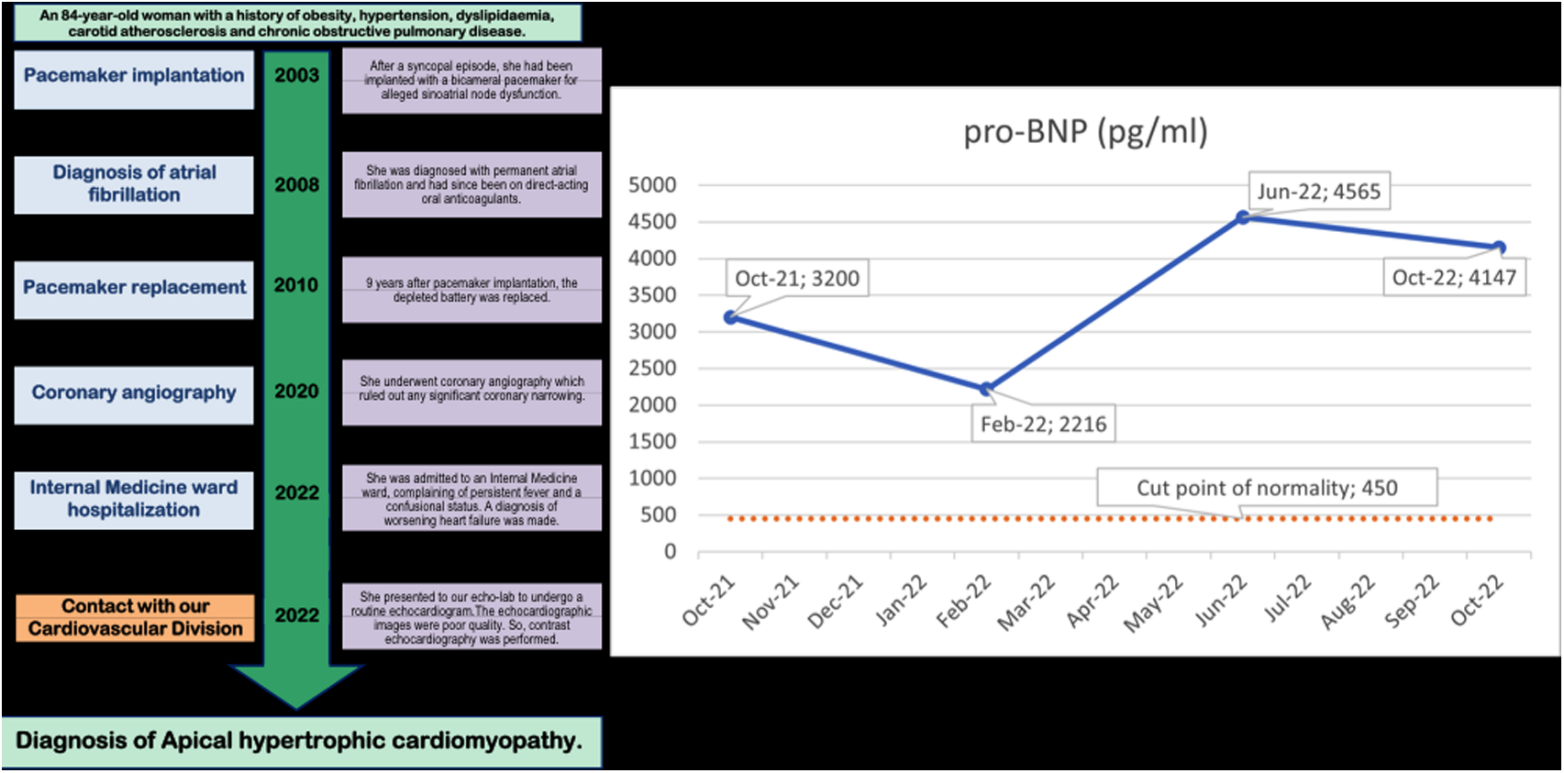
Timeline of clinical events in the 20 years before the diagnosis (on the left) and Pro-BNP values in the previous year (on the right). The Pro-BNP values are mildly but consistently abnormally high in each of the 4 evaluations.

Blood tests showed constant, modestly abnormal high pro-BNP (B-type natriuretic peptide) in the last year (Figure 1), which was systematically interpreted as chronic congestive heart failure caused by the hypertensive heart disease, with apparently isolated diastolic dysfunction. But this conflicted with the very mild hypertrophy of the LV. When she came to our attention for the echocardiographic evaluation her medical therapy included: apixaban 5 mg bis in die; pantoprazole 40 mg/die; bisoprolol 2,5 mg/die, furosemide 50 mg/die, canrenone 50 mg/die and digoxin 0,25 mg/die. Moreover, on the suspicion of ischemic heart disease, she had been taking rosuvastatin 10 mg/die, despite never suffering chest pain.

A timeline of the events occurring over the 20 years before the diagnosis of ApHCM is reported on the left side of the Figure 1.

### Physicals

The patient was overweight (BMI 35.11 kg/m^2^), with difficulty in walking. Physicals showed mildly increased systolic blood pressure (145/80 mmHg) (7), a well palpable apex beat in left lateral decubitus, substantially normal regarding amplitude and duration of the outward movement and without pre-systolic humps (but the patient was in atrial fibrillation [Afib]). The carotid amplitude pulse was normal but left the impression of a minimally brisk rate of rise. Jugular pulse inspection showed the absence of the x' descent, that was replaced by a significant x'v outward systolic wave followed by a very rapid y descent (secondary to significant tricuspid regurgitation). The hepato-jugular reflux (further jugular engorgement with enhancement of the top blood column after right upper abdomen compression lasting >30 s) and Kussmaul sign (increase of jugular top blood column in inspiration) were positive secondary to elevated systemic venous pressure. Auscultation elicited an irregular heart rate, the first heart sound with variable intensity and a soft systolic murmur on the xyphoid area, whose intensity remained constant after a long pause.

### EKG, and chest x-ray

**Figure 2:**
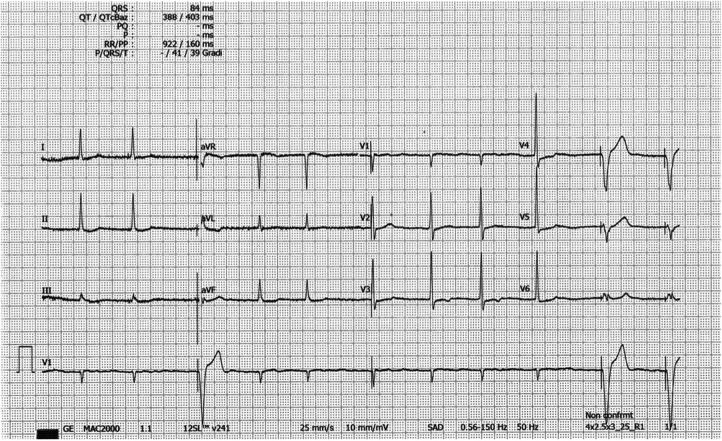
EKG tracing with no typical giant negative T waves. The rhythm is Afib; The R voltage of the QRS is modestly elevated in V3-V5 with only a shallow negative T wave in V3. Paced QRS complex. EKG = electrocardiogram; ApHCM = apical hypertrophic cardiomyopathy; Afib = atrial fibrillation.

The resting electrocardiogram showed a paced rhythm alternating with rare native QRS complexes; the native QRS complex showed no typical for ApHCM giant T waves but only minimal atypical repolarization anomalies of the QRS in V4 (Figure 2).

Chest x-ray showed a slightly enlarged heart with partial calcification of the aortic arch and a thickened thoracic aorta.

### Transthoracic echocardiography

**Figure 3:**
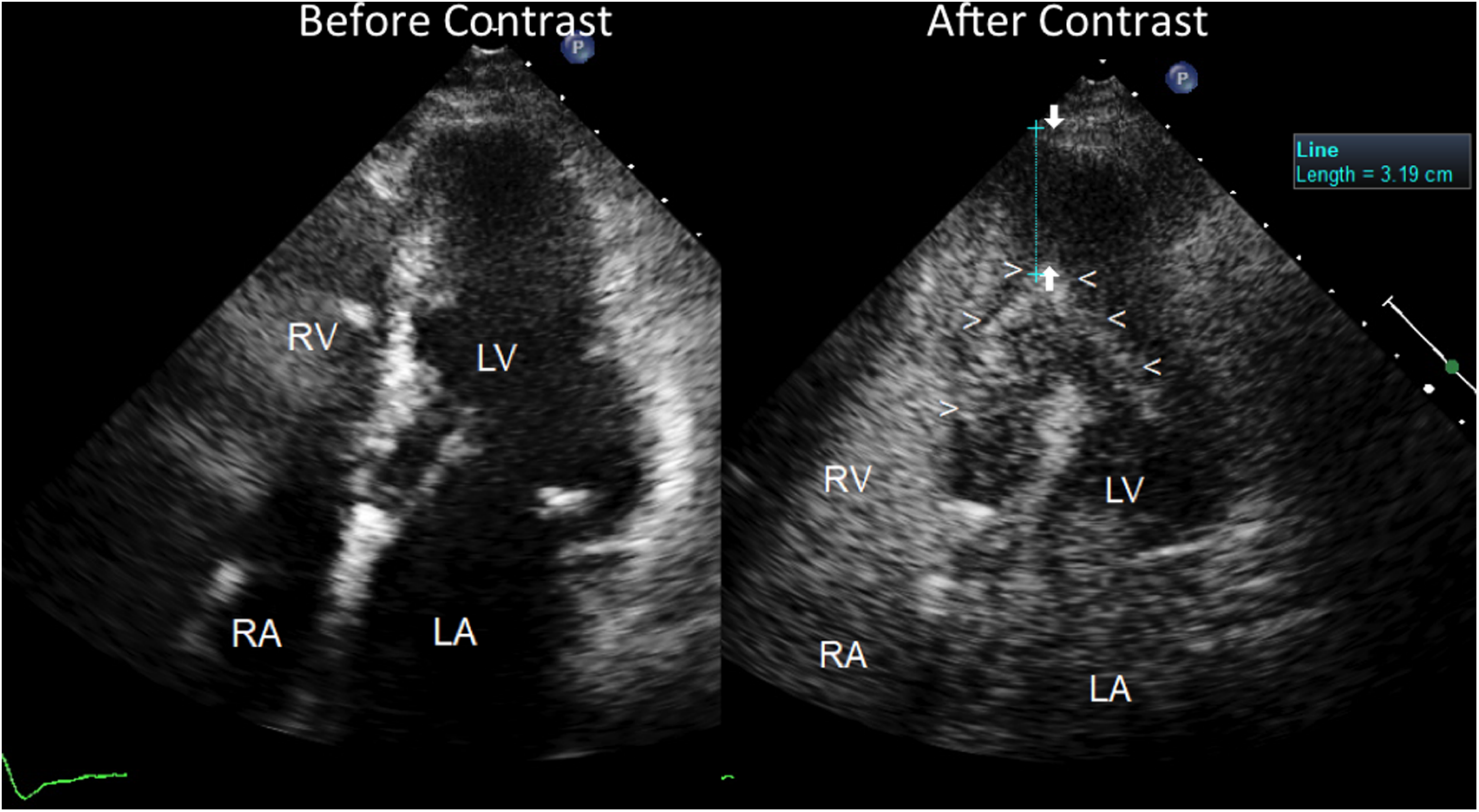
Severe apical left ventricular hypertrophy, as assessed by contrast enhanced transthoracic echocardiography. Four chamber view before (on the left) and after ultrasound contrast injection (on the right). Before contrast, the LV apical region does not show any clear delineation of the endocardial border but reveals a fairly homogeneous low backscatter echo-structure resembling a blood pool. No evident abnormality of the LV apical region can be noted but just poor quality imaging of the apical region with scarce delineation of the endocardium; the pace-maker leads can be noted in the right atrial cavity. On the contrary, after contrast injection (Sonoview® 1 cc in bolus) (on the right), a perfect delineation of the true apical endocardium is very clearly depicted as a hyper reflective line of backscatter (arrowheads). The severe hypertrophy of the LV apical wall can be noted and measured, as indicated by arrows (>3 cm thickness). The left atrium is dilated. The right atrial cavities are filled with contrast medium. LV = left ventricle; RV = right ventricle; RA = right atrium; LA = left atrium.

**Figure 4:**
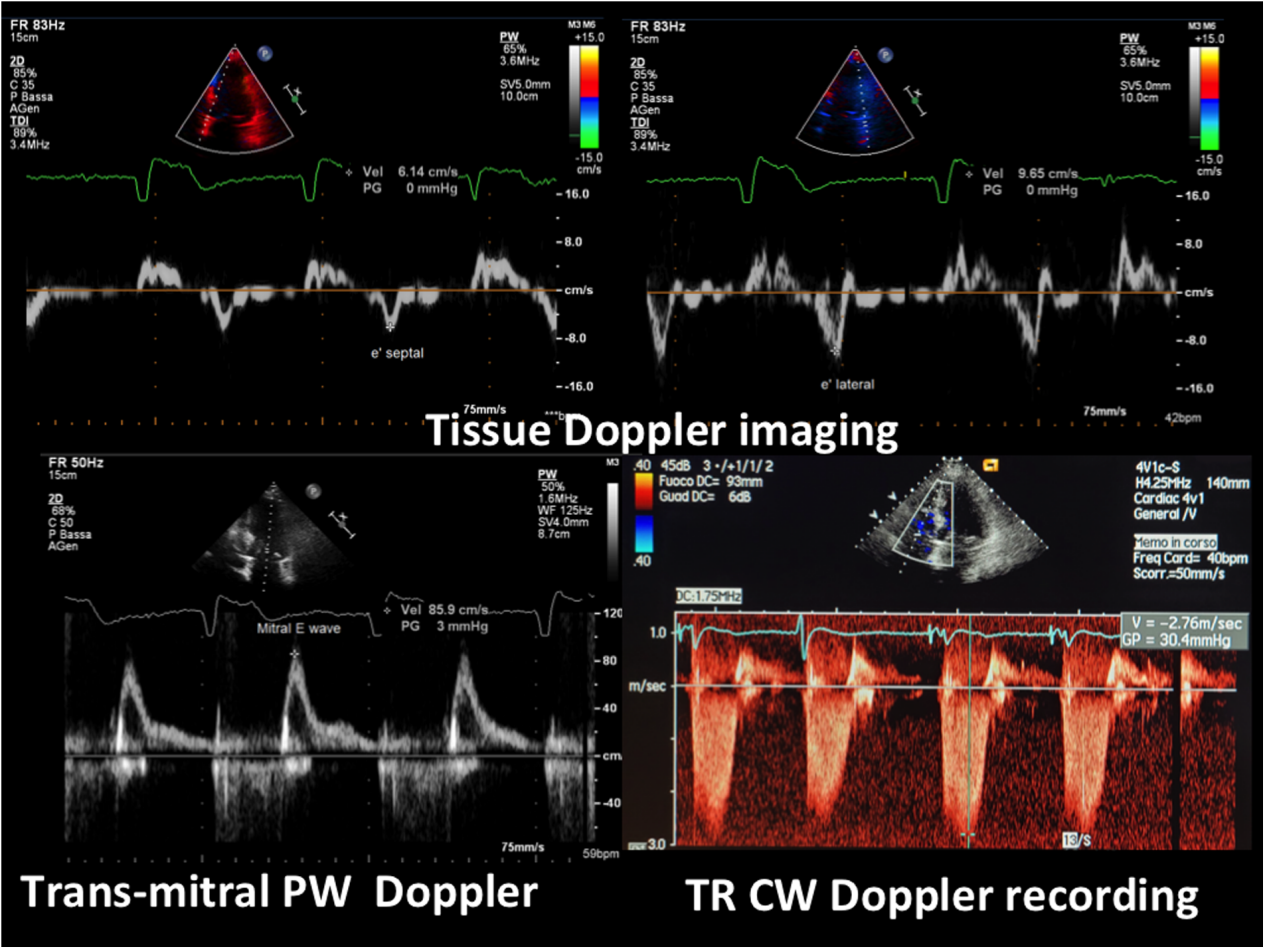
E/e’ and pulmonary pressure evaluation by transthoracic Doppler. At the top, tissue Doppler imaging of the movement of the septal (on the left, e’ septal wave) and of the lateral mitral annulus (on the right, e’ lateral wave); at the bottom (on the left) transmitral blood flow Doppler recording (mitral E wave) (the A wave is lacking since the rhythm is atrial fibrillation). The E/e’ ratio is elevated, indicating a high mean left atrial pressure confirmed by the elevated pulmonary pressure, as assessed by the maximal velocity of the TR jet (>2.7 cm/s) (bottom right). PW = pulsed Doppler; CW = continuous Doppler; TR = tricuspid regurgitation.

The echocardiogram performed in our echo Lab was of poor quality, especially the apical window projections. It showed a mild thickening of the inter-ventricular septum (septal thickness: 12 mm; posterior wall thickness = 11 mm), with a dilated left atrium (antero-posterior LA diameter = 41 mm) and hyper-normal (74%) LV ejection fraction. Both ventricles were apparently of normal size (LV diastolic diameter: 42 mm, RV basal diameter 37 mm). There was no evidence of wall motion abnormalities or dynamic left ventricular outflow tract obstruction. At first glance, the LV apical cavity did not show any abnormality, but endocardial delineation at the apex level was absent (Figure 3). In addition, there was no colour filling of the LV apex cavity even with the reduced Nyquist limit, that favours the mapping of even a markedly slow velocity flow. LV systolic function appeared hyper- normal, since at end systole, the systolic cavity was almost obliterated (the ejection fraction was not measured because of the scarce endocardial border delineation at the apex). Contrarily, LV diastolic function assessment was abnormal, revealing a possible mild elevation of left atrial and LV filling pressure. In fact, we found an elevated E/e' ratio (>12) along with a borderline venous flow abnormality (borderline rapid deceleration of the D wave), associated with left atrial dilation and mild-moderate pulmonary hypertension (45 mmHg) (normal upper value 25–30 mmHg) (Figure 4).

Regarding valve function, we found moderate-severe tricuspid regurgitation (Figure 4) and mild mitral regurgitation.

The right ventricle showed mild ventricle contractile dysfunction: mildly dilated secondary to significant tricuspid regurgitation but with normal (not hyper-normal) systolic function. The inferior vena cava and the hepatic veins were dilated, with reduced vena cava inspiratory collapse indicating pressure elevation, and in the supra-hepatic veins, systolic retrograde flow (only during inspiration) as the effect of significant tricuspid regurgitation. The significant tricuspid valve incompetence appeared to be the combination of primary tricuspid dysfunction (leaflets damage) induced by the pace-maker ventricular wire that could damage the valve at the valve crossing points, along with annular dilation induced by the mild-moderate pulmonary hypertension.

Owing to an uncertain picture of the LV apex function and morphology, since the endocardial border was not visible at that level, we decided to use contrast enhancement, using Sonoview® contrast medium, Bracco Diagnostics: each ml of the dispersion contains 8 *μ*l of sulphur hexafluorane in the microbubbles, equivalent to 45 micrograms (8). The duration of enhancement after a bolus is longer than 3 min, so we injected 1 ml of bolus solution, allowing the duration of the enhancement to cover 3 min of scanning. The contrast very clearly depicted the endocardial border of the apex cavity as a hyper reflective line that delimited a restricted apex cavity, partially filled with contrast medium. The restricted LV apical cavity showed a typical “ace-of-spades"-like configuration, with severe apical hypertrophy (maximal thickness = 3.6 cm) (Figure 3) and hyper-normal ejection fraction (74%). Thus, a reliable diagnosis of apical HCM could be made.

## Discussion

The main particularity of this case is that a diagnosis of ApHCM was not made for decades, and the unexpected progressively worsening HF was not appropriately explained. A unique convergence of several factors regarding clinical, EKG and echocardiography findings conspired to hamper the proper diagnosis, and misdiagnosis possibly hastened the disease progression owing to inappropriate drugs prescription and the lack of a more profound modulation of appropriate nutrigenomic and the associated epigenetic interventions (4, 5). Nonetheless, the patient was an octogenarian, confirming the relatively benign disease expression of this variant of hypertrophic cardiomyopathy (1).

Considering the difficulty in diagnosing ApHCM, we believe the main problem lies in executing and interpreting the echocardiograms (9). The non typical EKG findings may have contributed to the misdiagnosis (10). Indeed typical “giant” T waves are found in only 47% of patients with ApHCM and they are also less frequently observed in patients outside Japan (11). Finally, poorly accurate bedside investigations, especially before Afib onset (even if not specific) may also have contributed to the delayed diagnosis (12).

Echocardiography. In the literature, descriptions of the numerous caveats in the execution and interpretation of echocardiograms in this situation are lacking, especially in patients with a poor ultrasound window. In interpreting LV function and morphology during echo, the first step should be to check the endocardial border delineation, in particular at the level of the apex region. It is well known that the LV endocardial border can be incompletely delineated, especially in poor windows, since in this view the ultrasound beam is parallel to the endocardium and, therefore, fails to reflect enough ultrasound for effective visualization of the endocardial surface (13). During the scanning, the rapid sequence of the frames can give the false impression that the endocardial border is there and that the poor quality images are the cause of the poor endocardial definition. However, the bottom line is that all cases with poor endocardial border definition at the LV apex must be further investigated by contrast echocardiography, especially in difficult windows (8). A survey reported that in 31.7% of cases, echocardiography initially failed to diagnose ApHCM, later found at cardiac magnetic resonance imaging (CMRI) (9). In situations of unclear LV endocardial delineation, in particular at the apex, contrast echo is mandatory (14). That is the main indication for using contrast medium to enhance LV endocardial border delineation by echocardiography. CMRI can have possibly useful application for the diagnosis of ApHCM (15). However CMRI may implies additional risks, time delays, and costs (16). In particular a major drawback of CMRI is that it has got genotoxic effects as demonstrated by the significantly higher level of DNA double-strand breaks measured in human lymphocytes after exposure even with a 1.5 T machine as compared with pre exposure level (17). This cancerogenic effect is compounded by the possible gadolinium induced nephrogenic systemic fibrosis (18). Contrast echocardiography on the other hand is totally safe, so repeatable, showing the same diagnostic potential as CMRI to evaluate LV wall thickness and function with very favorable cost-effective analysis (16). So it is a preferable test for assessing hypertrophic cardiomyopathy and in general LV function and LV wall thickness in difficult patients. Moreover in ApHCM there is the need to assess coronaries, task that can be accomplished with enhanced echo Doppler that allows direct, functional evaluation of coronary stenosis in the left main and the whole left anterior descending coronary artery along with the integrative evaluation of coronary flow reserve in the distal LAD (19, 20); coronary stenosis detection with this functional approach appears even better than the morphologic approach by coronary computed tomography as preliminary demonstrated (21).

Another important aspect of our case is the presence of significant LV diastolic dysfunction, that went unexplained and was wrongly attributed to hypertensive heart disease. The evaluation of diastolic dysfunction in Afib can be challenging (22, 23). In our case unequivocal findings were present: firstly, dilation of the LA, that was the reason for the precocious Afib (Figure 3) associated with an E/e' > 12 (Figure 4) and secondarily, mild-moderate pulmonary hypertension (45 mmHg systolic) (Figure 4). Pulmonary venous flow Doppler recording, although extremely important for assessing LV diastolic function, is less reliable because atrial contraction is lacking in Afib (24).

The E/e' is a clear, validated index reflecting even in atrial fibrillation patients the increase of mean atrial pressure when the ratio gets higher than 12 (22, 23); it is expressed by the mitral E wave peak velocity that is normalized by the mitral annulus protodiastolic descent (e', evaluated by tissue Doppler imaging). As a consequence of elevated LV diastolic and left atrial pressure, the patient developed secondary pulmonary hypertension (Figure 4). The elevated pulmonary pressure was accurately evaluated by means of tricuspid regurgitation maximal velocity, a very reliable, validated Doppler finding (25). Then pulmonary hypertension either created or worsened the tricuspid regurgitation by means of tricuspid annulus dilation. Any increase in right ventricle afterload is handled by progressive dilation of the ventricle and this implies tricuspid annulus dilation owing to the specific anatomic features of that annulus (26).

This elevated left atrial pressure was also the reason for the persistently elevated proBNP in the previous year, as reported (27) (Figure 1). This was compounded by the dilation and elevated pressure in the right atrium secondary to the tricuspid regurgitation.

Over the years the patient was treated for heart failure symptoms, mainly with diuretics (Furosemide), at increased dosage to 50 mg/day after the last episode of heart failure. Neither angiotensin converting enzyme (ACE) inhibitors nor angiotensin II receptor blockers nor aldosterone inhibitors were prescribed. However, by reducing the circulating blood volume the use of loop diuretics can stimulate the renin angiotensin system and both directly and indirectly (by renin and angiotensin), the sympathetic outflow, especially without any ACE inhibition (28). The ensuing hormonal activation can further stimulate LV hypertrophy and myocardial fibrosis. Hypertension (the patient was moderately hypertensive), together with elevated circulating aldosterone, are associated with stimulation of cardiac fibroblasts and resultant augmented fibrosis in the hypertrophic tissue structure of the LV (29).

It is ironic that even though ACE inhibition drugs are widely used in hypertension, they were not used when specific ACE inhibition was potentially needed and worth trying (30). On the contrary, she was prescribed Digoxin for several years, that possibly further worsened the LV diastolic dysfunction (31).

Moreover, the LV diastolic dysfunction could have accelerated progression of the apical LV hypertrophy through stimulating the immune system, creating a vicious circle and worsening the disease. Left ventricular diastolic dysfunction, in fact, has been associated to the release of pro-inflammatory cytokines, prolonged hypoxemia, and excessive activation of neuroendocrine and autonomic nerve function, further aggravating the hormonal storms that foster myocardial hypertrophy and fibrosis (32). Experimental evidence has shown that blockade of interleukin 6, a typical inflammatory cytokine, attenuates left ventricular hypertrophy (33).

Finally, the patient was moderately overweight and this, with the ensuing hyperinsulinemia, could have further fueled the progression of the LV hypertrophy (34).

In conclusion, accurate and timing diagnosis with contrast echocardiography can prompt more precocious interventions in order to possibly prevent or delay the onset of the ApHCM complications; strain-independent factors (fibrosis and hypertrophy) are the more important target of the therapeutic interventions: weight reduction in obese patients with appropriate diet and normalization of hyperglycemia along with blood pressure normalization may reduce LV fibrosis/hypertrophy by several mechanisms (34) including modulating genes expression by a nutrigenomic effect (4); then ACE inhibition pharmacologic treatment could also possibly reduce myocardial fibrosis (35); other ancillary interventions are to avoid drugs that has got inotropic effect like digitalis and *implantable cardioverter-defibrillator* application (when indicated) that act on life threatening arrhythmias. Moreover, since ApHCM less frequently exhibits clear genetic familiarity, both European Society of Cardiology and American Heart Association HCM guidelines provide no ApHCM specific genotyping or family screening recommendations. Nevertheless, our opinion is to recommend serial cardiological evaluations, including EKG and echocardiography integrated with contrast enhancement in case of poor LV endocardial delineation, for the first-degree relatives of the patient, and to intensify follow-up in case of symptoms or apparent abnormalities thanks to the harmless effect of ultrasound energy.

## Conclusion

ApHCM can be a challenging diagnosis. Contrast echocardiography must always be applied in cases of poor delineation of the LV apical endocardial border at baseline echocardiography. Timely detection and appropriate lifestyle intervention might reduce the severity of LV hypertrophy and minimize and delay HF related symptoms and arrhythmias. The prognosis is seen to remain relatively benign after long term follow-up (20 years).

## Data Availability

The raw data supporting the conclusions of this article will be made available by the authors, without undue reservation.
